# The bifunctional dihydrofolate reductase thymidylate synthase of *Tetrahymena thermophila *provides a tool for molecular and biotechnology applications

**DOI:** 10.1186/1472-6750-6-21

**Published:** 2006-03-20

**Authors:** Lutz Herrmann, Ulrike Bockau, Arno Tiedtke, Marcus WW Hartmann, Thomas Weide

**Affiliations:** 1 Cilian AG, Johann-Krane Weg 42, D-48149 Münster, Germany; 2 Institut für allgemeine Zoologie und Genetik, Universität Münster, Schloßplatz 5, D-48149 Münster, Germany

## Abstract

**Background:**

Dihydrofolate reductase (DHFR) and thymidylate synthase (TS) are crucial enzymes in DNA synthesis. In alveolata both enzymes are expressed as one bifunctional enzyme.

**Results:**

Loss of this essential enzyme activities after successful allelic assortment of knock out alleles yields an auxotrophic marker in ciliates. Here the cloning, characterisation and functional analysis of *Tetrahymena thermophila's *DHFR-TS is presented. A first aspect of the presented work relates to destruction of DHFR-TS enzyme function in an alveolate thereby causing an auxotrophy for thymidine. A second aspect is to knock in an expression cassette encoding for a foreign gene with subsequent expression of the target protein.

**Conclusion:**

This system avoids the use of antibiotics or other drugs and therefore is of high interest for biotechnological applications.

## Background

*Tetrahymena thermophila *is a ciliated eukaryotic unicellular organism belonging to the regnum of protozoa and bearing two nuclei, a transcriptionally silent, diploid germline micronucleus (MIC) and a transcriptionally active, polyploid somatic macronucleus (MAC)[1]. In 1923, when Nobel Laureate Andre Lwoff succeeded in growing *Tetrahymena *in pure culture, the basis for exploiting this alveolate as a model organism was laid. Milestone discoveries made in *T. thermophila *are the discovery of dynein motors[2], telomeres[3], RNA-mediated catalysis[4], telomerase[5] and the function of histone acetyltransferases in transcription regulation[6]. Within the last decades molecular biological techniques have been developed to alter *T. thermophila*'s genome and proteome: DNA transfection methods range from microinjection[7] and electroporation[8] into the MAC to biolistic bombardment of MIC and MAC[9]. Episomal plasmids based on an rDNA-replicon are available[10], as well as knock out/-in techniques based on homologous recombination[11,12].

On protein level heterologous expression of related species has been performed[13,14] and also endogenous proteins were silenced by a novel antisense-ribosome-technique[15]. The biotechnological potential of *T. thermophila *has been proven in numerous publications, demonstrating fast growth, high biomass, fermentation in ordinary bacterial/yeast equipment, up-scalability, existence of cheap and chemical defined media [16-18].

Although known and analysed for decades, only a few markers have been described for *T. thermophila*. So far there are ribosomal point mutation mediated resistances, a plasmid based neomycin resistance[19] and a beta-tubulin selection marker making use of an inducible promoter in combination with mutated tubulins being resistant or sensitive to the mitotic drug taxol[20]. Yet no true auxotrophic marker is available that permits selection without the use of antibiotics or drugs.

Critical enzymes in pyrimidine biosynthesis are dihydrofolate reductase (DHFR) and thymidylate synthase (TS). DHFR catalyses the production of tetrahydrofolate from dihydrofolate; TS is in charge of transferring a methyl-group from N5, N10-methylene-tetrahydrofolate to dUMP thereby generating dTMP and tetrahydrofolate. These enzymes being crucial for pyrimidine synthesis have been used as auxotrophic markers in various systems by targeted gene disruption but also a number of inhibitors (antifolates) have been developed as anti-cancer drugs[21]. In animals, fungi and eubacteria the DHFR and TS gene are separately translated, whereas plants, alveolata and euglenozoa have a bifunctional fusion gene with both enzyme activities combined in one protein ("DHFR-TS").

The occurrence of the bifunctional enzyme in *T. pyriformis *has been postulated in 1984 and 1985 but no functional or even molecular biological analysis had been performed. A partial amino acid sequence of DHFR-TS of a non determined "*T. pyriformis*-like strain" has been published in 2002[22], but this work is lacking any proof of linkage to the described partial cDNA to enzyme function. Here we present a first characterization of the *T. thermophila *DHFR-TS gene including gene structure and functional data on the enzyme and data on *in vivo *function. For the first time we show that the *T. thermophila *DHFR-TS locus provides an auxotrophic marker system that enables monitoring of allelic assortment processes. As PCR based approaches always have to cope with wildtype alleles present in the MIC, the DHFR-TS auxotrophy system is able to deliver direct proof of the allelic assortment to be completed: Only in the case of all wildtype alleles in the MAC having been substituted by the knock out construct the auxotrophy will occur. Combining this methodology with a knock in, a new, stable and useful strain to express recombinant enzymes or proteins has been generated.

## Results

*Tetrahymenathermophila *is a free-living ciliated protozoan that has become increasingly interesting as an excellent expression system. It is one of the best characterised unicellular eukaryotes and its genome has been sequenced in its entirety (The Institute for Genomic Research[23]). These features form the basis for using *T. thermophila *in future biotechnology applications. However, appropriate marker systems that are essential tools for genetic manipulations are limited. The enzymes dihydrofolate reductase (DHFR) and thymidylate reductase (TS) play a crucial for pyrimidine synthesis, therefore both enzymes have been used as auxotrophic marker-systems in various species by targeted gene disruption. The aim was to establish such a DHFR marker system for *T. thermophila*. Interestingly, the bifunctional DHFR-TS has been as taxonomic tool to set up phylogenetic trees for years. These approaches led to a very rudimental DHFR-TS amino acid fragment sequence of a non determined "*T. pyriformis*-like strain". By using this incomplete information and data from the entire *T. thermophila *genome it was possible to determine the whole sequence of the bifunctional DHFR-TS MAC gene in *T. thermophila*. The *T. thermophila *DHFR-TS gene and its flanking regions were amplified by using the primer pairs DHFR 5'1F NotI, DHFR 5'2R BamHI for the 5'region non coding region, the primer pair DHFR 3'1F XhoI: 5, DHFR 3'1R Acc65I for the 3'region and the primers DHFR CDS-F, DHFR CDS-F for amplification of the coding region.

### The *T. thermophila *DHFR-TS gene structure

**Figure 1:**
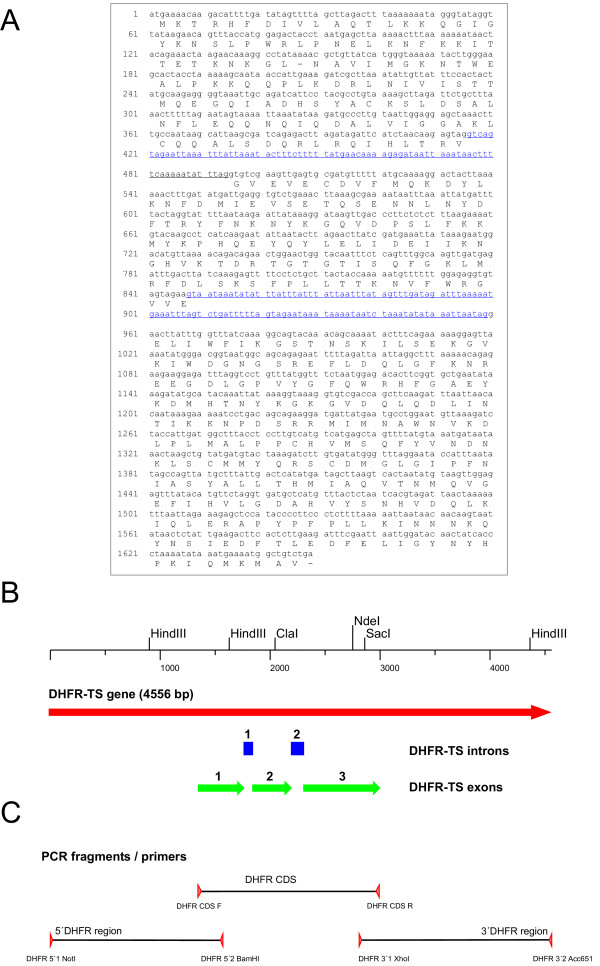
**Genomic structure of *T. thermophila *bifunctional DHFR-TS enzyme**. **A: **The DNA sequences of the gene and the cDNA as well as the deduced amino acid sequence are shown on the left (introns underlined, blue). **B: **The DHFR-TS gene structure of *T. thermophila *consists of three exons (green) and two introns (blue). **C:**Overview of primer pairs to amplify DNA for homologous integration and to amplify the CDS.

The comparison of the amino acid sequences of related species to the translated coding region of the DHFR-TS structure gene revealed the corresponding cDNA (see figure 1). Alignment with the cDNA with the gene revealed the intron exon boundaries. Figure 1 summarises the results and shows the MAC gene structure with two introns, the corresponding DNA sequence and the amino acid sequence of the *T. thermophila *DHFR-TS.

### Sequence alignment of the *T. thermophila *DHFR-TS enzyme to other alveolates

**Figure 2:**
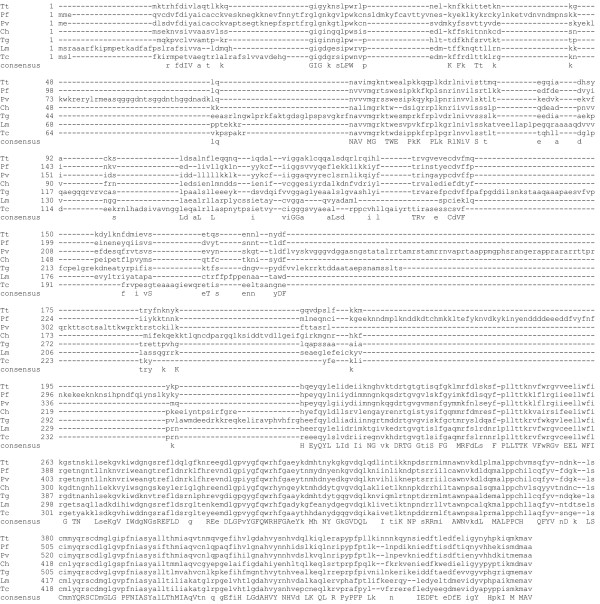
**Comparison of the *T. thermophila *DHFR-TS bifunctional enzyme to other protozoa**. Amino acid sequence comparison of *Tetrahymena thermophila *(Tt), *Plasmodium falciparum *(Pf), *Plasmodium vivax *(Pv), *Cryptosporidium hominis *(Ch), *Toxoplasma gondii *(Tg), *Leishmania major *(Lm) and *Trypanosoma cruzi *(Tc) DHFR-TS bifunctional enzyme.

The alignment of the DHFR-TS between the alveolates *Tetrahymena thermophila*, *Plasmodium falciparum*, *Plasmodium vivax*, *Cryptosporidium hominis *and *Toxoplasma gondii *(Tt, Pf, Pv, Ch, Tg in figure 2) illustrates a 50–60% degree of identity between the proteins. This is also the case if the ciliate DHFR-TS is compared to the analogous enzyme of *Leishmania major *and *Trypanosoma cruzi *(Lm, Tc in figure 2). This is almost due to the highly conserved thymidylate synthase part of the bifunctional enzymes. A highly conserved histidine is found at position 198, suggesting that the amino acids 198–485 represent the thymidylate synthase part of the bifunctional enzyme. Much more heterogeneity is found in the N-terminal part of the bifunctional enzyme that consists of the DHFR part and a linker site. The alignment reveals that almost all enzymes bear one or more individual, non-homologue amino acid stretches (figure 2).

### Proper integration of the knock out construct into the DHFR-TS locus and auxotrophy for thymidine

**Figure 3:**
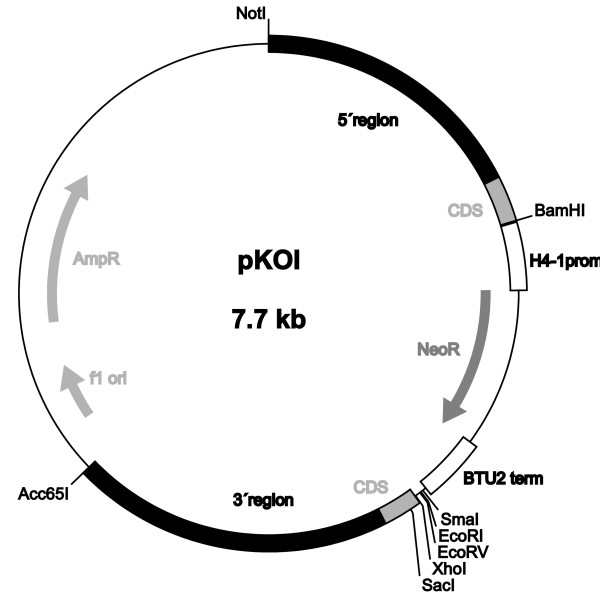
**pKOI: The basis for a DHFR-TS knock out construct**. Figure 3 shows the knock out construct used for a targeted knock out of the DHFR-TS gene in *T. thermophila*. It consists of 3' and 5' flanking regions of the *T. thermophila *DHFR-TS gene and parts of its coding sequence (CDS), disrupted by a functional neomycin cassette conferring resistance to paromomycin. For more details refer to material and methods section.

In order to verify that only one DHFR-TS activity is present in *T. thermophila *and that the sequence given in figure 1 corresponds to this activity a construct for knocking out the DHFR-TS gene was made. By inserting the flanking regions of the DHFR-TS gene into a pBS II SK backbone we created a plasmid for stable integration into the ciliate genome. The *neo2 *cassette of pH4T2[19] was used to monitor the successful uptake of this plasmid by selection against paromomycin. Figure 3 shows that the *neo2 *cassette is flanked by the ~1.5 kb fragments of the 5' and 3' parts of the non-coding regions of the DHFR-TS gene, respectively. Because the pBS backbone lacks an appropriate origin of replication, paromomycin resistant *T. thermophila *clones argue for proper homologous recombination in the DHFR-TS gene locus.

**Figure 4:**
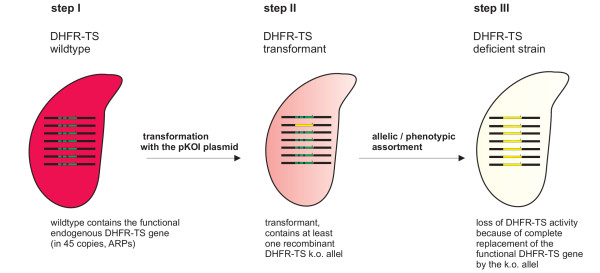
**Generation of DHFR-TS deficient strains by allelic assortment**. Wildtype strains are transfected with pKOI. In one copy of the 45 ARPs the endogenous DHFR-TS gene is substituted by the knock out construct (step 2). By amitotic division of the MAC and increasing selection pressure clones will arise, that have sorted out all endogenous DHFR-TS genes and retain only recombinant and defect DHFR-TS genes (step 3).

**Figure 5:**
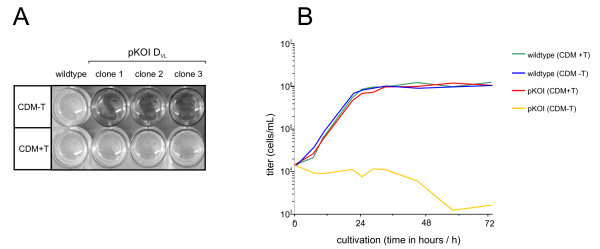
**Growth of DHFR-TS deficient *T. thermophila *compared to wildtype**. **A: **Selection of DHFR-TS knock out cells by growth on thymidine. *T. thermophila *cells with disrupted DHFR-TS gene (clone 1–3) do not grow without the presence of thymidine (CDM-T), whereas wildtype cells do. Addition of thymidine to the medium (CDM+T) recovers growth. **B: **Growth kinetics of DHFR-TS knock out cells compared to wildtype cells in media with or without thymidine show, that the knock out strain (pKOI) is growing as fast as wildtype cells on thymidine supplemented media (CDM+T). Knock out cells die without thymidine present (CDM-T). The curves are calculated by mean values of at least three independent experiments.

The most convincing evidence for the correct integration into the DHFR-TS locus is a loss of the DHFR-TS activity in the transformed strain. However, in the case of *T. thermophila *this requires the complete replacement of all chromosomal DHFR-TS wildtype alleles (~45 ARPs) by the ARPs that includes the knock out cassette. We achieved this by allelic assortment, a phenomenon summarised in figure 4. This allelic or phenotypic assortment is based on randomised distribution of the MAC chromosomes units (ARPs) during mitosis. In order to force the assortment process into the desired direction – namely into the recombinant resistance gene – the transformed cells were cultivated for at least 3–4 weeks using increasing concentrations of the drug paromomycin. Single clones were isolated and tested for DHFR-TS deficiency by using a minimal chemical defined medium (CDM) with (+T) and without thymidine (-T). It could be shown that DHFR-TS knock out clones are real auxotrophic strains. The mutants are able to grow in CDM with thymidine like wildtype strains or strains with an incomplete allelic assortment. In CDM lacking thymidine they are unable to grow (figure 5).

### Functional addition of an expression cassette to the knock out construct

The *knockout *of the endogenous DHFR-TS gene of *T. thermophila *also provides the possibility to *knockin *a further foreign gene that can be expressed heterologously in the DHFR-TS knock out strains. Therefore we named our constructs pKOI (pKOI: knock out and knock in). To illustrate this knock out/-in concept we constructed the pKOI D_VL _plasmid. It consists of a pKOI backbone with an additional expression cassette that encodes the first 115 amino acids (aa) of the precursor sequence of the PLA_1 _gene and the mature human DNase I (aa 23 to 281). The PLA_1 _pre/pro-peptide (aa 1 to 110) has significant similarity to members of the cathepsin L family and mediates secretion into the medium[24]. The five additional amino acids (aa 111 to 115) should ensure an optimal cleavage of the pro PLA_1_-DNaseI fusion protein by endogenous pro-peptidases. In contrast to the *neo2 *cassette the expression of the ppPLA_115_-DNase I fusion protein is regulated by the inducible *MTT1 *promoter[25]. The inducible system was selected because it allows a clear discrimination between the DNase activity of heterologously expressed recombinant human DNase I and the basal activity due to at least two endogenous DNases. The transformation, selection of positive clones and the directed allelic assortment were done as described in the material and methods section.

**Figure 6:**
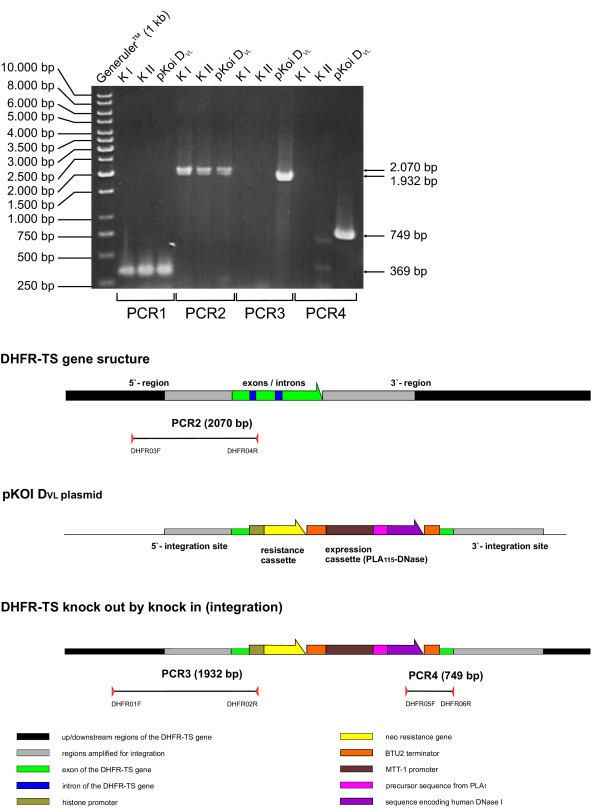
**Proper integration of DHFR-TS knock out construct**. This figure illustrates the PCR approach to determine that the knock out/-in construct has integrated into the DHFR-TS gene locus. Three different cells were tested: KI is a wildtype control, KII are cells transfected with a plasmid carrying only the disrupting cassettes but no DHFR-TS gene sequences and pKOI D_VL _are cells transformed with the pKOI D_VL _plasmid. PCR1 is a control reaction amplyfing 369 bp of the beta-hexosaminidase gene. PCR2 is to detect endogenous DHFR-TS (note that in the pKOID_VL _cells there still is a wildtype gene in the MIC). PCR3 only yields PCR-product for correctly integrated pKOI D_VL _DNA. PCR4 shows that the full-length expression cassette has integrated. The first scheme illustrate the overall structure of the DHFR-TS gene, exons are coloured in green and introns in blue. The parts of the non-coding 5' and 3' regions of the DHFR-TS structure gene that we used as integration sites in the pKOI/pKOI D_VL _plasmids are coloured in grey. The next scheme gives on overall structure of the pKOID_VL _expression construct that was used for transformation and integration into the MAC. The third scheme illustrates the disruption of the endogenous DHFR-TS structure gene by proper integration of the resistance and expression cassettes into the MAC.

**Figure 7:**
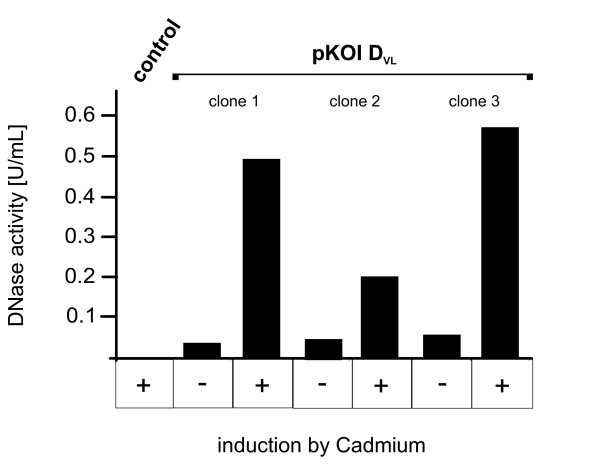
**Enzyme function of knock in target**. Supernatants of three clones transformed with pKOI D_VL _were assayed for DNase activity. Only heavy metal induced cells (+) show high levels of DNase activity. Transformed, but non-induced cells show a slightly elevated enzyme activity compared to wildtype cells due to low basal promoter activity.

**Figure 8:**
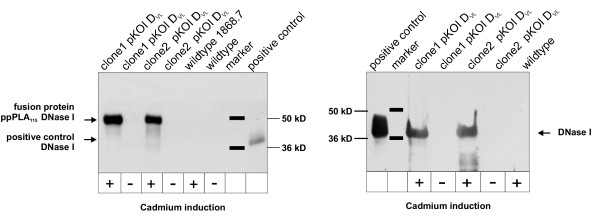
**Expression of knock in target**. Western blots show expression of recombinant human DNase I: Only transformed and heavy metal induced cells (+) show strong signals due to anti-DNase I antibodies. The intracellular PLA_1_-DNase-fusion protein is visualized on the left blot by samples of cell lysates. Bands are running at higher molecular weight than the mature and processed positive control due to the pre/pro-peptide. On the right, supernatants were subjected to Western blot; the size of the secreted protein of *T. thermophila *argues for a correct processing when compared to the positive control.

Furthermore, we tested the correct and complete integration of both expression cassettes (*neo2 *and the DNase I) in the DHFR-TS locus by a PCR approach (figure 6). In order to demonstrate the pKOI concept, cells of these DHFR-TS knock out strains carrying the ppPLA_115_-DNase expression cassette were treated with and without Cadmium. Only induced strains showed an elevated DNase activity in the supernatant (Figure 7). To confirm this enzymatic data a specific antiserum against human DNase I was used to analyse the cell extracts and the supernatant of these human DNase expressing DHFR-TS knock out strains by Western blot. The results illustrate that the DHFR-TS knock out strains are capable of expressing and secreting the functional recombinant human protein (Figure 8).

## Discussion

The ciliated protozoan *T. thermophila *features two nuclei, a somatic macronucleus (MAC) and a genetic micronucleus (MIC) offering different possibilities of manipulating the organism's properties (for a short and comprehensive review see[26]).

It is well known that altering the cell's phenotype ultimately needs direct or indirect (by MIC transformation and conjugation) genetic engineering of the vegetative MAC. The first approaches for heterologous expression were done by using plasmids that use the vast amplification of the rDNA gene during *anlagen*/MAC development. However, the episomal presence of these plasmids depends on the drug concentration and the plasmid may recombine homologously and non-directionally into endogenous rDNA units.

Stable integration of expression cassettes into diploid MIC can be achieved by biolistic bombardment. After conjugation of two different mating types the old MACs disappear and new ones form that carry the new information derived from the recombinant MIC. The whole process follows the statistics of the Mendelian genetics. The advantage of this approach is that one obtains stable clones that maintain the genetic properties and that can be crossed via classical genetics to combine various properties of different *T. thermophila *strains. This approach is elaborative and time consuming. Furthermore, it has been shown recently that scan RNAs (snRNA) derived from the old MAC play an important role in DNA elimination during the development of the somatic MAC from the germline MIC (see [27] for review). The primary sequence of these small RNAs explains how the parental MAC epigenetically controls the genome rearrangement in the new MAC. In the case of stable MIC transformants this RNAi-like mechanism may cause partial deletion of foreign expression cassettes in the developing new MAC [28].

Therefore instead of episomal transformation by rDNA based plasmids or the stable transformation of the MIC we used a shortcut by combining MAC transformation with allelic assortment. This combination has several advantages. Firstly, the MAC transformation is much more efficient because there are at least about 45 potential integration sites per gene locus. This increases the probability of integration to at least one order of magnitude. Secondly, not only conjugating but also non-conjugating and therefore defined strains can be transformed. This is very important, because it allows the stepwise improvement of strains by maintaining defined genetic properties. Thirdly after completed allelic assortment the use of any antibiotic is obsolete as wildtype alleles have vanished in a one-way-manner. This allows cheap cultivation at large scales for e.g. biotechnical production processes.

## Conclusion

In summary use of the DHFR-TS gene locus as a target for homologous genomic integration does not only provide a robust and simple marker system to assure complete allelic assortment of altered ARPs in the MAC but also the pKOI concept combining the DHFR-TS knock out with an additional knock in and subsequent expression of foreign genes.

## Methods

### Cells and cell culture

*Tetrahymena thermophila *strains B 1868/4, B 1868/7 and B 2068/1 were cultivated in skimmed milk medium[29] in SPP (0.5% proteose peptone, 0.5% yeast extract, 0.1% ferrous sulphate chelate solution and 1% glucose) or in CDM medium[18].

### Amplification of the DHFR-TS gene of *T. thermophila*

The DHFR-TS cDNA, gene including 5'and 3' flanking sites can be amplified using the following primer pairs. Nucleotides in small letters encode sites for restriction endonucleases. Amplification of the DHFR-TS 5' flanking region:

DHFR 5'1 F NotI: 5'-cccgcggccgcACAGAGTTAATGGAAATGGAGC-3'

DHFR 5'2R BamHI: 5'-gggggatccATATTTAAGCGATCTTTCAATGG-3'

Amplification of the DHFR-TS cDNA and gene with introns:

DHFR CDS-F:

5'-cgcGAATTCATGAAAACAAGACATTTTGATATAGTTTTAGC-3'

DHFR CDS-R:

5'-gcgCTCGAGTCAGACAGCCATTTTCATTTATATTTTAGGG-3'

Amplification of the DHFR-TS 3'flanking region:

DHFR 3'1F XhoI: 5'-gggctcgagATGCTCATGTTTACTCTAATCACG-3'

DHFR 3'1R Acc65I: 5'-gggggtaccAGTAAAAATAGAGTAGAAGGAG-3'.

### Construction of plasmids

#### Construction of pKOI

The pKOI (knock out/-in) plasmid was constructed as follows: As backbone for selection and propagation in *E. coli*. the pBlueScript II SK plasmid was used. The 1.5 kb 5'-DHFR-TS integration site was amplified using the primer pair DHFR 5' 1 F NotI and DHFR 5' 2 R BamHI cloned into pBS II SK by using NotI and BamHI sites. Next the 1.4 kb paromomycin selection cassette from the pH4T2 *(neo2) *was cloned into the intermediate pBS IISK by BamHI and SmaI sites. Finally, the 3'-DHFR-TS integration site was amplified by primers DHFR 3' 1 F XhoI and DHFR 3' 1 R Acc65I and cloned by using the XhoI and Acc65I sites to finish the DHFR-TS knock out cassette. The SacI site of the pBS II SK backbone had been destroyed by site directed mutagenesis to facilitate the use of the endogenous SacI site in the 3'-DHFR-TS integrating sequence and the XhoI site as unique cloning site in pKOI. The whole pKOI basis vector is ~7.7 kb in size and contains a multiple cloning site (figure 3).

#### Construction of pKOI D_VL_

The unique XhoI and SacI sites were used to insert the ppPLA_115_-DNase knock in expression cassette (~2.5 kb). This cassette encodes a fusion protein of the first 115 aa of the endogenous PLA_1 _precursor and the aa 23–281 of the mature human DNaseI, flanked by a ~1 kb *MTT1 *promotor active sequence and the ~0.4 kb *BTU2 *terminator, leading to a ~10.2 kb vector. To ensure proper translation of this fusion protein a codon optimised synthetic human DNase I gene was used (submitted at BMC Biotechnol.)

The junction sequence between the *MTT1 *promoter and the precursor sequence of PLA_1 _is given with the sequence ATGgatatcAAC, using a EcoRV site (gatatc) between the initial ATG of the *MTT1 *gene and the second codon (AAC) of the PLA_1 _precursor sequence. The cDNA that encodes the ppPLA_115_-DNase I fusion protein ends with the TGA stop codon followed by a BglII site (agatct). Therefore the "knock in" cassette (generated in an intermediate vector) offers a modular structure that allows the simple replacement of the promoter (by XhoI/EcoRV), the coding sequence (by EcoRV/BglII) and the terminator DNA sequences (by BglII/SacI). Further details of the primers and constructs are available from the authors.

### Transformation of pKOI plasmids (biolistic bombardment)

We used conjugating cells, as well as vegetative, growing or stationary *T. thermophila *strains. The transformation of the *T. thermophila *cells was performed as previously described in[9].

### Selection, allelic assortment and DHFR-TS knock out assay

*T. thermophila *cell proliferation assay: For the first ca 16 h after biolistic bombardment transformants were grown in skimmed milk medium. After that transformed cells were grown on SPP medium with in increasing concentrations of paromomycin (from 100 μg/mL to 1000 μg/mL) to support the allelic assortment process. After 3–4 weeks each clone was cultivated on CDM replica plates with or without thymidine (10 mg/mL). Functional DHFR-TS knock out clones are only able to grow in CDM medium supplemented with thymidine. The viability of the DHFR-TS knock out strains was monitored by determining the growth kinetic. The complete integration of the DHFR-TS knock out and DNase I knock in cassette was confirmed by PCR using the following primers:

DHFR01F: 5'-CTTTTTAACAGCCTGCTGCTCG-3'

DHFR02R: 5'-GATTTTGATGCTTCAATAAGGTTG-3'

DHFR03F: 5'-TTATTTGTTTTATCATAGTGGAAAAGG-3'

DHFR04R: 5'-CAGACACCTCAATCATATCAAAG-3'

DHFR05F: 5'-GGTCCTCCATCAGATTGTGG-3'

DHFR06R: 5'-CGCGTCGAGTCAGACAGCCATTTTCATTTA-3'

Hex01F: 5'-ATGCAAAAGATACTTTTAATTACTTTC-3'

Hex02R: 5'-TATATTTTAGGAATGTTGTAATC-3'

A pH4T2 plasmid carrying the same *neo2 *and DNase I expression/secretion cassettes was used as PCR control.

### SDS-PAGE and Western blot

Aliquots of transformed cells and of SPP supernatants were resuspended in sample buffer and separated on 15 % SDS-PAGE. The gels were blotted onto nitrocellulose membranes and blocked in PBS containing 0.05 % Tween 20 and 5 % skim milk (PBS-TM). The expression of recombinant human DNase I in transformed Ciliates was detected by two specific anti sera from rabbit against human DNase I (antigen: recombinant human DNase I, Pulmozyme, Roche). Both sera detected the recombinant DNase I antigen. The serum was used in a 1:500 dilution in PBS-TM. After washing with PBS/T and applying an HRP-conjugated anti rabbit serum. The blots were developed by using chemiluminescence.

### DNase I activity assay

The methyl green based DNase activity assay was performed as already published [30]. Samples were incubated at 37°C for 24 h on a microtiter plate. Absorbance was measured at 620 nm. Calibration of the assay was achieved by different amounts of defined DNase I Units of Pulmozyme from Roche (CHO derived) in each experiment and linear regression. These results combined with semi-quantitative Western blotting were used to calculate the specific activity of expressed DNase I.

### Sequence alignments

DHFR-TS sequences were aligned with Sci Ed Central's Clonemanger Professional Suite v6.0 using the BLOSUM 62 score matrix.

## Authors' contributions

LH evaluated the DHFR-TS targeting sequences, setup and cloned the pKOI vector system and participated in manuscript drafting. UB participated in performing the experiments (cloning of pKOI D_VL _expression constructs, transformation and screening of the ciliates, including monitoring of foreign gene expression) and in figure preparation. MWWH conceived of the study and participated in its design and coordination. TW participated in project conception, preparation of figures, did sequence alignments and was involved in manuscript drafting. All authors read and approved the final manuscript.
